# A randomized controlled trial of web-based cognitive behavioral therapy for severely fatigued breast cancer survivors (CHANGE-study): study protocol

**DOI:** 10.1186/s12885-015-1787-7

**Published:** 2015-10-23

**Authors:** H. J. G. Abrahams, M. F. M. Gielissen, M. M. Goedendorp, T. Berends, M. E. W. J. Peters, H. Poort, C. A. H. H. V. M. Verhagen, H. Knoop

**Affiliations:** Expert Center for Chronic Fatigue (ECCF), Radboud University Medical Center, PO Box 9101, 916, 6500 HB Nijmegen, The Netherlands; Department of Health Sciences, University Medical Center Groningen, University of Groningen, Groningen, The Netherlands; Department of Medical Oncology, Radboud University Medical Center, Nijmegen, The Netherlands

**Keywords:** Breast cancer, Survivor, Fatigue, Cognitive behavioral therapy, Web-based CBT, E-health, Randomized controlled trial, Study protocol

## Abstract

**Background:**

About one third of breast cancer survivors suffer from persistent severe fatigue after completion of curative cancer treatment. Face-to-face cognitive behavioral therapy (F2F CBT), especially designed for fatigue in cancer survivors, was found effective in reducing fatigue. However, this intervention is intensive and treatment capacity is limited. To extend treatment options, a web-based version of CBT requiring less therapist time was developed. This intervention is aimed at changing fatigue-perpetuating cognitions and behaviors. The efficacy of web-based CBT will be examined in a multicenter randomized controlled trial.

**Methods:**

In total, 132 severely fatigued breast cancer survivors will be recruited and randomized to either an intervention condition or care as usual (ratio 1:1). Participants will be assessed at baseline and 6 months thereafter. The intervention group will receive web-based CBT, consisting of three F2F sessions and maximally eight web-based modules over a period of 6 months. The care as usual group will be on a waiting list for regular F2F CBT. The total duration of the waiting list is 6 months. The primary outcome of the study is fatigue severity. Secondary outcomes are functional impairments, psychological distress and quality of life.

**Discussion:**

If web-based CBT is effective, it will provide an additional treatment option for fatigue in breast cancer survivors. Web-based CBT is expected to be less time-consuming for therapists than regular F2F CBT, which would result in an increased treatment capacity. Moreover, the intervention would become more easily accessible for a larger number of patients, and patients can save travel time and costs.

**Trial registration:**

Dutch Trial Registry - NTR4309

**Electronic supplementary material:**

The online version of this article (doi:10.1186/s12885-015-1787-7) contains supplementary material, which is available to authorized users.

## Background

Worldwide, breast cancer is the most common malignancy in women. About 1.7 million new cases were diagnosed in 2012 [1]. In the last decades, survival rates have been improved due to early detection by screening programs and advances in oncological treatments [2, 3]. Since the number of breast cancer survivors increases, concerns are raised about their long-term well-being. After completion of curative cancer treatment, side-effects can become chronic. One of these persistent side-effects is cancer-related fatigue [3]. The National Comprehensive Cancer Network defined cancer-related fatigue as “a distressing, persistent, subjective sense of physical, emotional and/or cognitive tiredness, related to cancer or cancer treatment, that is not proportional to recent activity and interferes with usual functioning” [4]. Once the malignancy is successfully treated, the fatigue is expected to decrease. Nevertheless, severe fatigue becomes a chronic condition in approximately one-third of breast cancer survivors [5–8].

### Interventions for fatigue in cancer survivors

Since persisting severe fatigue interferes with daily functioning and has profound effects on quality of life, it should not be left untreated [5, 9]. The evidence of available interventions was recently evaluated in a practice guideline of the American Society of Clinical Oncology [10]. It was concluded that there is evidence for the efficacy of physical and psychosocial interventions. Initiating or maintaining adequate levels of physical activity [11–19], (cognitive) behavioral therapy [20–25], and (psycho) educational interventions [20, 25, 26] can reduce fatigue. In addition, there is some evidence for the efficacy of mindfulness-based approaches [21, 27, 28], yoga [29, 30], and acupuncture [31, 32].

**Fig. 1:**
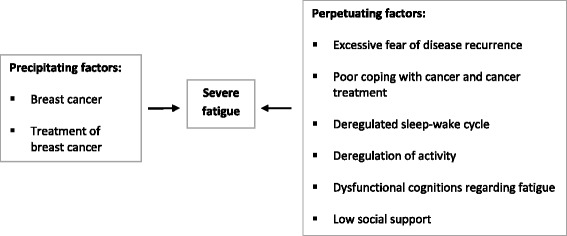
Explanatory model of the CBT protocol

The current study focuses on one of these evidence-based interventions: cognitive behavioral therapy (CBT). A CBT protocol for fatigue in cancer survivors with various tumor types was developed and tested in a randomized controlled trial (RCT) at our treatment center, the Expert Center for Chronic Fatigue of the Radboud university medical center (Radboudumc) [22]. This RCT showed that patients reported a clinically significant reduction in fatigue and functional impairments following CBT [22]. These effects were maintained at a 2-year follow-up [33]. The efficacy of the CBT protocol was recently replicated in a RCT of Prinsen et al. [34]. The CBT protocol is based on a model of precipitating and perpetuating factors of fatigue [22]. According to this model, the malignancy and its treatment are the precipitating factors that induced fatigue. However, other factors are responsible for the persistence of severe fatigue after cancer treatment [22]. These fatigue-perpetuating factors and the overall explanatory model are captured in Fig. 1. Each fatigue-perpetuating factor is addressed in a module of the CBT protocol, offered as regular face-to-face (F2F) therapy. However, this F2F CBT is intensive for both therapists and patients, since it consists of 12 to 14 F2F sessions over a period of 6 months. Therapists need to invest considerable time to deliver these sessions and a limited number of trained cognitive behavioral therapists provide this F2F therapy. Besides, patients need to travel to a treatment center to attend the sessions. The development of web-based CBT would reduce the therapist time needed to deliver the intervention and increase treatment accessibility for severely fatigued breast cancer survivors.

### Web-based CBT

The fast-growing field of e-health has created new possibilities in the development of web-based interventions. Web-based CBT has been developed and examined for a wide range of mental health problems, and so far, results are promising. Multiple studies have shown that web-based CBT can be effective in reducing mental health problems [35]. To extend treatment options for severely fatigued breast cancer survivors, we have developed a web-based version of our F2F CBT protocol for severely fatigued cancer survivors, named “On the road to recovery”*.* The efficacy of this intervention will be examined in a RCT, named “the CHANGE-study”.

### The right time to intervene

In our previous RCT’s examining regular F2F CBT for severely fatigued cancer survivors, the intervention was offered at least 1 year after completion of cancer treatment [22, 34]. However, recent research has shown that the level of fatigue does not decrease further after 3 months following curative cancer treatment [36], and fatigue-perpetuating factors can already be identified at 3 months following cancer treatment [37]. Therefore, it might be possible to treat fatigue in cancer survivors at an earlier stage. To examine if this is the case, the web-based CBT will be offered at least 3 months after completion of cancer treatment.

### Aims of the CHANGE study

1. To examine the efficacy of web-based CBT for severely fatigued breast cancer survivors on fatigue severity compared to care as usual.
2. To examine the efficacy of web-based CBT for severely fatigued breast cancer survivors on functional impairments, psychological distress, and quality of life compared to care as usual.
3. To examine if time since completion of cancer treatment moderates the efficacy of web-based CBT with respect to fatigue severity.

## Methods

The method section of this study protocol is written in accordance with the CONSORT statement for reporting parallel group randomized trials [38] and the CONSORT e-health criteria for reporting web-based interventions [39].

### Design

A non-blinded multicenter RCT (the CHANGE-study) will be conducted to evaluate the efficacy of web-based CBT compared to care as usual for severely fatigued breast cancer survivors.

### Recruitment

#### Referrals by medical professionals

Patients will be recruited by medical professionals (physicians and nurses) at the outpatient clinic of the departments of surgery and/or oncology of eight hospitals in the Netherlands (Radboudumc, Nijmegen; Canisius Wilhelmina hospital, Nijmegen; hospital Gelderse Vallei, Ede; hospital Bernhoven, Uden; hospital Pantein, Boxmeer; VieCuri medical center, Venlo; Elkerliek hospital, Helmond; Slingeland hospital, Doetinchem). Physicians and nurses will inform eligible patients about the study during regular medical follow-up consults and give them an information leaflet. If a patient agrees to be informed about the study by the researcher, the nurse practitioner will fill out a participation form and send it to the researcher (HA). Subsequently, the researcher will call the patient to give a detailed explanation about the study and to address questions.

As a second recruitment strategy, nurse practitioners from selected participating hospitals will identify cohorts of eligible patients through medical records. They will inform these cohorts about the CHANGE-study by mail. Patients will receive an information leaflet with an accompanying letter. In this letter, patients are asked to contact the researcher if they want to participate in the study.

#### Self-referrals

Patients will also be informed about the study by leaflets and notifications on social media of patients’ associations and the Radboudumc (e.g. Facebook and Twitter). Patients can complete a participation form, integrated in an informative website. Subsequently, the researcher will contact patients by phone to inform them about the study and to address questions.

### Participants

All patients who want to participate in the study will first be screened for eligibility. The in- and exclusion criteria are shown in Table 1. To verify the medical criteria (criterion 2, 3, and 4) of self-referrals, patients will send a copy of the most recent report of their medical follow-up examination to the researcher. The researcher will administer an online screening questionnaire to verify the other criteria. All patients will sign informed consent before filling out this online screening. The Checklist Individual Strength [40] will be used to screen for severe fatigue (criterion 6). The Beck Depression Inventory for Primary Care (BDI-PC) [41, 42] will be used to screen for a depressive disorder (criterion 9). If the score on the BDI-PC is ≥4, the researcher will administer the Depression module of the Mini-International Neuropsychiatric Interview (M.I.N.I.) [43] by phone to assess the presence of a major depression. If patients meet the criteria for major depression, they will be advised to contact their general practitioner for an appropriate referral.

**Table 1:** In- and exclusion criteria

Inclusion criteria
1) Women who are 18 years or older.
2) Treated for breast cancer with curative intent.
3) Breast cancer treatment (surgery, chemo- and/or radiotherapy) must be finished at least 3 months previously. There is no upper limit for the time since completion of cancer treatment. Patients who currently receive hormone and/or targeted therapy are eligible.
4) Disease-free at entry of the study, defined by the absence of somatic disease activity parameters.
5) Able to speak, read, and write Dutch.
6) Severely fatigued, defined by a score of ≥35 on the fatigue severity subscale of the Checklist Individual Strength.
7) Having access to a computer with internet.
Exclusion criteria
8) Presence of a co-morbidity that explains the presence of severe fatigue.
9) A depressive disorder, assessed with the BDI-PC and the M.I.N.I.
10) Current psychological treatment for a psychiatric disorder.
11) Current CBT for fatigue.

### Procedure

**Fig. 2:**
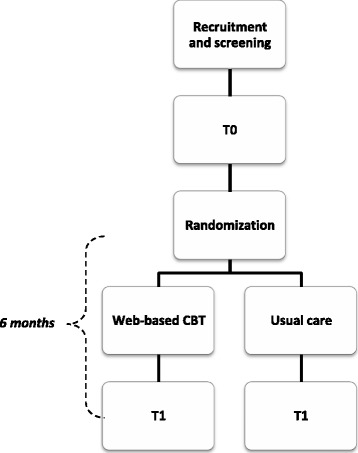
Overall study design. T0 = baseline assessment; T1 = second assessment

If patients are eligible and have signed written informed consent, they will start with a baseline assessment (T0). Following T0, participants will be randomized to either the intervention condition (web-based CBT) or the control condition (care as usual). After 6 months, all participants will be assessed again (T1). For participants assigned to the web-based CBT, this will be the post-intervention assessment. The overall study design is shown in Fig. 2. A test assistant will perform T0, T1 and the randomization.

### Randomization

Stratified randomization will be based on time since completion of cancer treatment (3 months up to 1 year versus ≥ 1 year) and type of referral (referrals by medical professionals versus self-referrals). After T0, randomization will be performed by a test assistant in the presence of the patient. A computerized randomization tool, built by an independent statistical expert, will be used to randomly allocate patients to either intervention or control condition. The allocation ratio will be 1:1 and block-randomization will be used with a block size of six. The test assistant, the researcher and the participants will be blinded to the allocation sequence. They will not be blinded for the randomization outcome, because this is not possible in psychological treatments.

### Intervention

#### Development

**Fig. 3:**
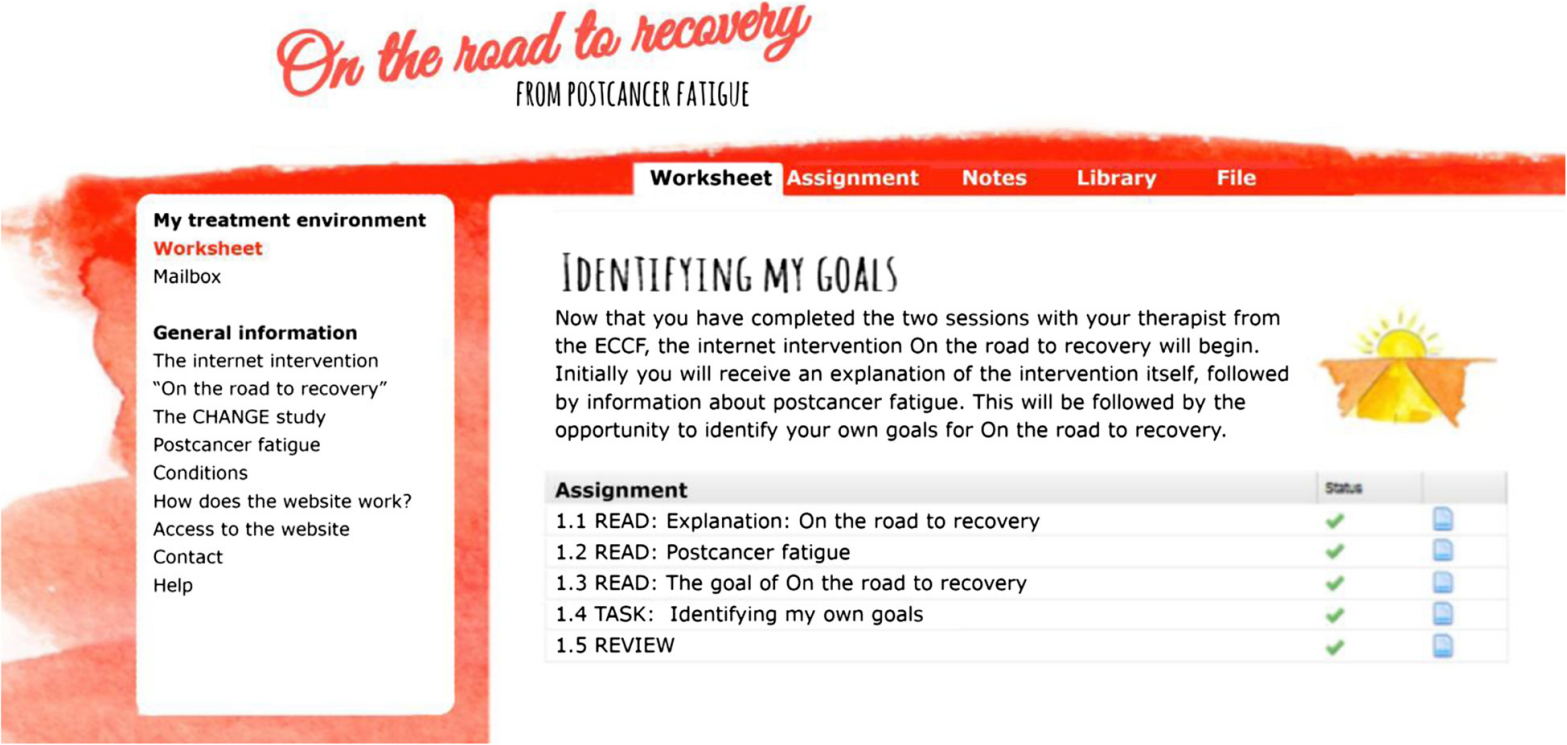
Screenshot of *On the road to recovery*

*On the road to recovery* is built in a web portal, designed with technical guidance from the Psychological and Psychiatric Care Innovation (Utrecht, The Netherlands) [44]. Experts in the field of fatigue in cancer survivors developed the content of this web portal. Trained, experienced cognitive behavioral therapists (HK, TB) and researchers (MGI, HA) wrote the texts and assignments. In total, the web portal consists of 13 texts and 26 assignments. A graphic designer developed the lay-out of *On the road to recovery*, and a videographer made 13 videos together with a therapist (HK) and the researcher (HA). These videos are integrated in the web portal. In the first video, a medical oncologist (SV) explains the rationale of the CBT. The other 12 videos are interviews of three cancer survivors. These patients are recovered from fatigue after receiving F2F CBT, and tell about their experiences with the CBT modules. A screenshot of the web portal is provided in Fig. 3. For this occasion, the text is translated into English.

#### Usability testing

Five severely fatigued breast cancer survivors, who were following F2F CBT, participated in a test pilot. The usability of the web portal was tested by using a “think aloud procedure” [45]. Participants were asked to think aloud while independently completing the modules. In the meanwhile, the researcher (HA) noted obstacles they encountered (i.e. usability problems and problems with text readability). Afterwards, all participants filled out a feedback form. They were asked about the sufficiency of information provided, text readability, and the lay-out and usability of the web portal. The findings of the usability testing were used to optimize the final version of the web portal.

#### Intervention condition: on the road to recovery

All participants in the intervention condition will follow *On the road to recovery,* a web-based version of the regular F2F CBT for severely fatigued cancer survivors. Participants will start with two F2F sessions with their therapist. In these sessions, the CBT model for fatigue in cancer survivors (Fig. 1) will be explained and a treatment plan will be made. Thereafter, participants will follow *On the road to recovery* online. The web-based CBT consists of eight treatment modules. All participants will start with setting their treatment goals *(module 1)*. Then, they will work on the fatigue-perpetuating factors that are applicable to them: (1) poor coping with breast cancer and breast cancer treatment; (2) high fear of cancer recurrence; (3) dysfunctional fatigue-related cognitions; (4) a deregulated sleep-wake rhythm; (5) a deregulated activity pattern; and/or (6) negative social interactions and low social support. Each of these six fatigue-perpetuating factors coincides with a treatment module *(module 2–7)*. At baseline assessment, it is decided which modules are relevant for each participant. Finally, all participants will complete the therapy by realizing their treatment goals *(module 8). On the road to recovery* is tailor-made. Assessment tools are used to assess which fatigue-perpetuating factors are present and to determine which treatment modules patients need to follow (Table 2). All treatment modules consist of three parts: psycho-education (“READING”), assignments in which participants work on fatigue-perpetuating factors (“DOING”) and a final assignment, in which participants evaluate their progress (“REVIEW”). The content of the eight treatment modules is described in more detail in Additional file 1.

**Table 2:** Tools to assess which treatment modules are indicated

Treatment module	Instrument [REF]	Outcome	Response format	Psychometric properties	Cut-off value
1. Coping with cancer and cancer treatment	Impact of Event Scale [62]	Intrusion and avoidance	4-point Likert scale, range 0–60	Cronbach’s α ranges between 0.87 and 0.96; adequate convergent validity [63]	Score ≥10 for each separate scale
2. Fear of cancer recurrence	Modified cancer acceptance scale [53]	Fear of disease recurrence	4-point Likert scale, range 3–12	N/A	Score ≥7
	Cancer worry scale [64]	Worries about the risk of developing cancer (again)	4-point Likert scale, range 8–32	Cronbach’s α = 0.87; good convergent and divergent validity [64]	Score ≥14 [64]
3. Helpful thinking	Modified causal attribution list [52, 65]	Somatic and non-somatic attributions	4-point Likert scale	Cronbach’s α ranges between 0.71 and 0.77 [65]	N/A
	Illness management questionnaire [65–67]	Focusing on symptoms	6-point Likert-scale, range 9–54	Cronbach’s α ranges between 0.85 and 0.93 [66]	Score ≥30
	Fatigue catastrophizing scale [68]	Catastrophizing in response to fatigue	5-point Likert scale, range 1–5	Cronbach’s α = 0.85 [68]	Score ≥2 (magnifying); score ≥7 (ruminating).
	Self-efficacy scale [33, 65]	Self-efficacy with respect to fatigue	4 point Likert scale, range 7–28	N/A	Score ≤19
4. Sleep-wake rhythm	Sleep-wake diary	Sleep-wake rhythm	Bedtimes and wake-up times of 12 consecutive days and nights	N/A	N/A
5. Activity regulation	An actometer, a motion-sensing device, worn to the ankle for 12 consecutive days and nights	Activity pattern (relatively active versus low active)	Average physical activity level (number of accelerations per 5 min period) [69]	Adequate reliability and validity [73]	N/A
6. Social support	Van Sonderen Social Support Inventory, subscales Interactions (SSLI) and Discrepancies (SSLD) [70]	Discrepancy between actual and desired social support	4-point Likert Scale, range 34–136	Cronbach’s α = 0.93 (SSLI); α = 0.95 (SSLD); good content validity [71]	Score ≥14 (SSLI); score ≥50 (SSLD)

Therapists will contact patients two-weekly by e-mail to give feedback on their progress and to answer questions. Therapists can also initiate video sessions with a secured video consultation system (Facetalk) [46]. These video sessions are in particular recommended for the modules “Fear of cancer recurrence” and “Coping with cancer and cancer treatment”. The guideline is to plan maximally two video sessions. The maximum duration of *On the road to recovery* is 6 months. Therapists will be blinded for the level of fatigue severity (primary outcome measure). Only after the post-treatment assessment (T1), they will be informed about the levels of fatigue severity on T0 and T1. The outcomes with respect to fatigue severity and other disabilities will be discussed with the participant in a final F2F session. In this session, the therapist and patient will determine if the patient is recovered from severe fatigue. If patients are not recovered from severe fatigue, F2F therapy will be offered outside the study context.

#### Treatment integrity

*On the road to recovery* will be given by licensed cognitive behavioral therapists. All therapists are experienced in working with the F2F CBT protocol for severe fatigue in cancer survivors. They will participate in a weekly supervision, in which cases are discussed in the presence of senior clinical psychologists (HK, TB). Changes in individual treatment plans will be made according to the study protocol and to the CBT principles for severely fatigued cancer survivors.

At the end of the study, a random 5 % of the e-mail messages send to the patients will be evaluated. An experienced clinician (HK) and researcher (HA) will determine whether the web-based CBT was delivered according to the predefined treatment protocol. To determine if web-based CBT is less time consuming than F2F CBT, therapists will register the invested time for each patient.

#### Control condition: care as usual

Participants in the control condition will be on a waiting list for regular F2F CBT for fatigue in cancer survivors. The total duration of the waiting list is 6 months. In this period, patients will receive care as usual. The usual care for breast cancer survivors in the Netherlands consists of follow-up examinations conform the Dutch guidelines for oncology care [47]. The frequency of these follow-up examinations depends on age, time since diagnosis and a possible BRCA1/2 mutation. In general, there will be a 3-month follow-up in the first year, a biannual follow-up in the second year, and an annual follow-up in the following years up to 5 years after diagnosis.

Recently, a guideline for the management of psychosocial distress in breast cancer survivors is implemented [48]. According to this guideline, psychosocial problems are identified and patients should be referred to specialized care providers. Participants may therefore be referred to other fatigue-oriented interventions during the study (e.g. psychosocial interventions, a rehabilitation trajectory, or physical therapy). At T1, all participants will be asked if they have received any treatment for fatigue during the study, and if so, they are asked to describe this treatment.

### Outcomes

#### Primary outcome

*Fatigue severity*, measured by the subscale Fatigue Severity (8 items, 7-point Likert Scale) of the Checklist Individual Strength (CIS) [49]. This subscale consists of eight items, scored on a 7-point Likert scale. The range of scores is 8 to 56, with a higher score indicating a higher level of fatigue. The cut-off score for severe fatigue is ≥35 [49]. The CIS has been established as a valid and reliable measure [50, 51], which showed sensitivity to detect change in previous studies investigating fatigue in cancer survivors [22, 33, 52, 53].

#### Secondary outcomes

*Functional impairments,* measured by the Sickness Impact Profile 8 (SIP) [54, 55]. This questionnaire addresses the level of disability in eight domains: alertness behavior, sleep/ rest, homemaking, leisure activities, mobility, social interactions, ambulation, and work. The weighted total score on these eight domains will be used to assess functional disability, with higher scores indicating more disabilities. The SIP is a reliable measure with sufficient content validity [56].

*Psychological distress*, measured by the total score on the Brief Symptom Inventory 18 (BSI-18) [57]. This multidimensional questionnaire consists of 18 items, scored on a 5-point Likert scale. The range of scores is 0 to 72, with a higher score indicating more psychological distress. The BSI-18 is a shortened version of the Symptom Checklist 90 (SCL-90) [58]. The BSI-18 has high levels of sensitivity and specificity [59].

*Quality of life*, measured by the European Organization for Research and Treatment of Cancer Quality of Life Questionnaire Core 30 (EORTC-QLQ-C30) [60]. This questionnaire consists of 30 items that cover five function scales (physical, role, cognitive, emotional and social functioning), three symptom scales (fatigue, pain, and nausea and vomiting), and a global health and quality of life scale. All scales are scored on a 4-point Likert scale. The EORTC-QLQ-C30 has been established as a valid and reliable measure [61].

#### Other variables

Demographic variables will be assessed by using a self-report questionnaire at T0. The instruments used to determine the relevant fatigue-perpetuating factors are shown in Table 2.

### Power

The sample size calculation is based on the guidelines of Borm et al. for analysis of covariance (ANCOVA) in RCT’s [72]. A clinically relevant difference of six points is expected for the primary outcome (fatigue severity subscale of the CIS) between the intervention and control condition. This difference is based on a study of Knoop et al. [73], in which the efficacy of a minimal intervention for patients with chronic fatigue syndrome was examined [73]. A minimum number of 60 patients per condition would be needed for a *t*-test with an alpha of 0.05, a two-sided significance level and a power of 0.85. According to Borm et al. [72], this number of patients needs to be multiplied by a “design factor” to calculate the needed sample size for an ANCOVA [76]. This factor is one minus the squared correlation coefficient between the baseline and outcome measure of fatigue severity. In our previous study examining the efficacy of F2F CBT for fatigue in cancer survivors, the correlation of the baseline and outcome measure was 0.36 [22, 33]. This leads to a factor of 0.87 (1 - .36^2^ = 0.87). Thus, the minimal number of patients in each condition is 53 (60*0.87 = 52.2) . The drop-out rate in our first RCT examining F2F CBT for fatigue in cancer survivors was 13 % [22, 33]. In the current study, patients might experience less support from their therapist in the web-based CBT. Therefore, the drop-out in the current RCT is estimated to be 50 % higher than in the first RCT (1,5*13 = 19,5 %) Therefore, a margin of 19,5 % for drop-out is added to the minimal number of 53 patients per condition. This results in a sample size of 132 severely fatigued breast cancer survivors.

### Intended statistical analyses

The primary objective of the study is to examine the effects of web-based CBT on reducing fatigue severity compared to care as usual. Therefore, an analysis of covariance (ANCOVA) will be used with the CIS-fatigue score at T1 as dependent variable, the CIS-fatigue score at T0 as covariate and condition as fixed factor [39]. The clinical importance of the treatment effect will be determined. Differences between the intervention and control condition on the amount of change in fatigue severity will be calculated on T0 and T1. Clinically meaningful change will be defined as a reliable change index of more than 1.96 and a decrease of the fatigue level to a normal range (i.e. a score of <35 on the fatigue severity subscale of the Checklist Individual Strength). The effects of web-based CBT on the secondary outcomes of the study (functional impairments, psychological distress and quality of life) compared to care as usual will be determined with ANCOVA’s. For each secondary outcome measure, an ANCOVA will be performed with the score of the outcome measure at T1 as dependent variable, the score at T0 as covariate and condition as fixed factor. The third objective of the study is to examine if time since completion of cancer treatment moderates the effects of web-based CBT. This will be analyzed with an ANCOVA with time since completion of cancer treatment (3 months-1 year versus ≥1 year) as covariate. The CIS-fatigue score at T1 will be the dependent variable, and the fatigue score at T0 will be the second covariate. All data analyses will be based on intention to treat. Missing values on primary and secondary outcome measures will be replaced with multiple imputation using fully conditional specification with at least five imputations. In case of statistically significant differences, a sensitivity analysis will be performed, based on different assumptions about the values of missing data.

### Ethical approval

This study has been reviewed and approved by the Medical Ethical Committee of the Radboudumc (reference no. 2013/167). The study has also been approved by the local ethical committees of each participating hospital (Radboudumc, Canisius Wilhelmina hospital, hospital Gelderse Vallei, hospital Bernhoven, hospital Pantein, VieCuri medical center, Elkerliek hospital and Slingeland hospital). The study is registered in the Dutch Trial Registry (reference no. NTR4309, date registered: December 6, 2013).

## Discussion

The CHANGE study will examine the efficacy of a web-based version of an evidence-based CBT protocol for severe fatigue in breast cancer survivors. The efficacy of the intervention on fatigue, functional impairments, psychological distress and quality of life will be examined as well. Web-based CBT has several advantages over F2F CBT; (i) e-mail contacts are expected to be less time consuming for therapists than F2F contacts, which would result in an increased treatment capacity; (ii) the intervention becomes more easily accessible for a larger number of patients, and (iii) the burden for patients can be reduced, because they can save travel time and costs to the treatment center. Besides, patients can work on the intervention at their own pace, at any preferred time.

After completion of the patient inclusion, the CHANGE study will be extended to form a non-inferiority trial. In this trial, stepped care will be compared to F2F CBT for severely fatigued breast cancer survivors. The first step in the stepped care condition will be web-based CBT. If patients are not recovered from severe fatigue after completion of web-based CBT, additional F2F CBT sessions will be offered. We will examine whether the effects of stepped care on fatigue severity are noninferior to regular F2F CBT after a waiting period. We will also determine whether stepped care requires less therapist time than regular F2F CBT. The non-inferiority trial is registered in the Dutch Trial Registry (reference no. NTR5179).

In conclusion, if web-based CBT is effective, it would provide an additional treatment option that is easily accessible for breast cancer survivors suffering from severe fatigue.

## Additional file

Additional file 1:
**Overview of the treatment modules of** ***On the road to recovery.*** (DOCX 64.3 kb)

## Abbreviations

ANCOVA: Analysis of covariance
CBT: Cognitive behavioral therapy
CHANGE study: Cognitive behavioral therapy, an online intervention for fatigued breast cancer survivors
CIS: Checklist individual strength
ECCF: Expert Center for Chronic Fatigue
F2F: Face-to-face
RCT: Randomized controlled trial
