# Supplementary material for: A randomized controlled trial of web-based cognitive behavioral therapy for severely fatigued breast cancer survivors (CHANGE-study): study protocol
Source: BMC Cancer. 2015 Oct 23;15:765. doi: 10.1186/s12885-015-1787-7 (PMC4619089; doi:10.1186/s12885-015-1787-7)
Supplement: Additional file 1: — Overview of the treatment modules of On the road to recovery. (DOCX 64.3 kb) [file 12885_2015_1787_MOESM1_ESM.docx]

**Additional file 1** Overview of the treatment modules of *On the road to recovery*
The CBT protocol for fatigue in cancer survivors is aimed at changing fatigue-related cognitions and behaviors. *On the road to recovery* consists of eight treatment modules. All patients will start with module 1 (goal setting) and finish with module 8 (realizing of goals). The intermediate six modules coincide with six fatigue-perpetuating factors, and can differ between patients depending on their baseline assessment. Assessment tools are used to determine which factors are applicable (Table 2). Each patient will work on at least one fatigue-perpetuating factor. All treatment modules are illustrated in Figure 4. In total, patients will follow from three up to eight treatment modules:

***Module 1: Goal setting***This module starts with an explanation of the web portal and the rationale of *On the road to recovery*. The cognitive behavioral model of fatigue in cancer survivors (Figure 1) is explained to patients. This model assumes that the fatigue is induced by the cancer and cancer treatment, but other factors cause the fatigue to persist. Subsequently, patients are asked to set concrete treatment goals. The overall goal of the intervention is no longer being severely fatigued and no longer being disabled by fatigue. Concrete goals are the activities patients would do (and do not do now), if they were no longer limited by severe fatigue.
 ***Module 2: Coping with cancer and cancer treatment***Being treated for cancer can be a traumatic event. If patients keep reliving or actively avoiding memories of this period in their life, they might suffer from posttraumatic symptoms that can perpetuate fatigue. The aim of this module is to help patients with the processing of their experiences. To this end, patients will first complete a targeted writing assignment. They will write about the events, their experiences and its impact from breast cancer diagnosis up to now [74]. After writing it down, patients will repeat reading their story until they no longer feel distressed when thinking of the cancer and cancer treatment. Talking about their story with their therapist (using Facetalk), their spouses or with significant others can be part of this process as well.
 ***Module 3: Fear of cancer recurrence***Fear of disease recurrence is normal after completion of cancer treatment. It is also normal that anxious thoughts increase in particular situations, like upcoming medical follow-up examinations. However, in some patients, fear of cancer recurrence is continuously and excessively elevated. These heightened levels of fear can perpetuate severe fatigue. In this module, patients will first get insight in the triggers of their anxiety. They will rank several situations that can provoke anxiety, and define their coping strategies in these situations. Then, patients will be helped to identify the cognitions underlying their fear. They will define what they are scared of, and the extent to which this corresponds with reality. If needed, patients are advised to talk with their physician to get insight in their actual risk of cancer recurrence. Finally, patients will learn to adopt helpful cognitions that can decrease their worrying. An example of a helpful cognition is: “It makes no sense to worry about the cancer coming back. This does not help me and only makes me feel bad”.
 ***Module 4: Helpful thinking***Dysfunctional fatigue-related cognitions can perpetuate severe fatigue. Examples are catastrophizing (i.e. having a highly negative orientation towards fatigue), a low self-efficacy (i.e. not feeling able to influence fatigue) and somatic attributions (i.e. attributing the fatigue mainly to the cancer and cancer treatment). These cognitions make patients feel like they have no control over their fatigue. In this module, patients will first assess their thoughts when feeling tired, and identify the subsequent feelings, behaviors and its consequences. Then, they will learn to replace dysfunctional thoughts with more realistic, helpful cognitions that can increase their self-efficacy. An example is: “I accept that I am tired, that is just how it is NOW. It does not have to stay like this, I am able to do something about the fatigue.”
 Another part of this module is learning to focus less on fatigue. Patients will learn how to shift their attention, for example by focusing on other activities or on the environment. Patients are advised to stop using fatigue as an indicator for what they can and cannot do, and no longer talk about their fatigue.

***Module 5: Sleep-wake rhythm***
Irregular sleep patterns are common in fatigued cancer survivors and can perpetuate severe fatigue. Keeping irregular bedtimes and wake-up times, and lying down or sleeping during the day can lead to a disrupted circadian pattern. In this module, a consistent sleep-wake pattern will be established. Patients will be temporarily asked to get up and go to bed at fixed times each day. They will also be asked not to sleep or lie down during the day. In this way, patients can (re)set their “biological clock”. Advices for adequate sleep-hygiene practices are given (i.e. adopt a regular going-to-bed ritual and avoid drinking any caffeinated drinks or alcohol before going to sleep).  ***Module 6: Activity regulation***After completion of cancer treatment, activity patterns can be deregulated. The activity patterns of all participants will be measured and divided in one of two categories:

1. *Relatively active:* these patients have fluctuating activity levels with bursts of activities followed by periods of inactivity (“all-or-nothing behavior”).
2. *Low active:* these patients have a continuous low level of physical activity. This may be habit, or patients may avoid activities out of fear of getting tired.

Both activity patterns can perpetuate severe fatigue. Relatively active patients will first learn to evenly distribute their activities, leaving sufficient space for unforeseen circumstances. Subsequently, they will gradually increase their (physical) activity level. Patients choose a physical activity that they can perform daily (walking or cycling). They gradually and systematically build up the duration of this physical activity. Low active patients will immediately start with this graded activity program. When patients are able to increase their physical activity level, their self-efficacy with respect to fatigue and being active often increases as well. This module finishes with optional assignments for building up mental and social activities, and resumption of work (if applicable).  ***Module 7: Social support***Severely fatigued breast cancer survivors can experience negative social interactions regarding their fatigue, like overly concerned responses or a lack of understanding of significant others. In this module, patients will learn how to communicate about their fatigue with significant others. They will also learn how to be more assertive, for example by setting clear limits concerning the information they want to share. Some patients might still expect the same amount of support from their environment as during their illness. When the expectations of patients with regard to social support are unrealistic, patients will learn to adopt a different attitude towards their environment and to modify their expectations. ***Module 8: Realizing goals***
When patients have finished building up their physical activity level, they are advised to start realizing the treatment goals they set in the first module. They will make an action plan and realize their pre-set goals step-by-step. Another part of this module is letting go of the regular sleep-wake rhythm and even distribution of activities. In this way, patients learn how to cope with disruptions in their sleep-wake rhythm and activity pattern. Finally, patients will evaluate the overall progress they have made during the treatment program *On the road to recovery*. Part of this evaluation is to determine if they consider themselves as recovered from severe fatigue.

**
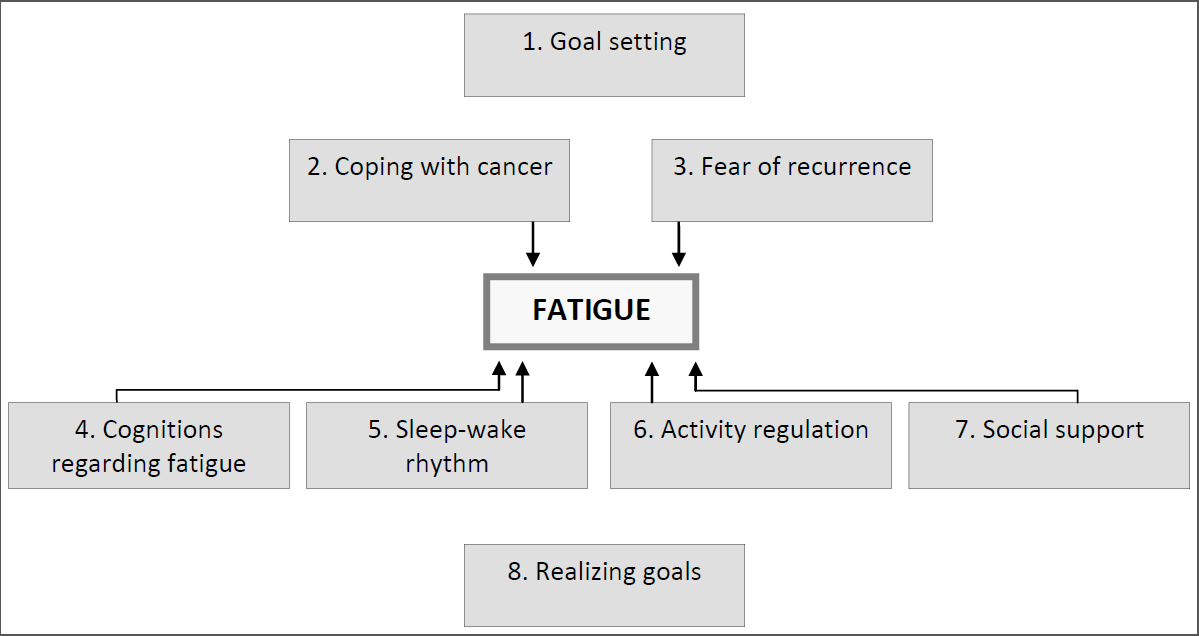
**

**Figure 4** Treatment modules of *On the road to recovery.*
